# High Prevalence and Regional Heterogeneity of Canine *Ancylostoma* spp. in Ecuador: A Systematic Review and Meta-Analysis and Its Potential One Health Implications

**DOI:** 10.3390/ani16142230

**Published:** 2026-07-18

**Authors:** Rommel Lenin Vinueza, Mateo Mantilla, Jorge Miño, Renato León, Roberto D. Coello-Peralta

**Affiliations:** 1 Escuela de Medicina Veterinaria, Universidad San Francisco de Quito (USFQ), Quito 170901, Ecuador; rvinueza@usfq.edu.ec (R.L.V.); mmantillab@estud.usfq.edu.ec (M.M.); .; 2 Laboratory of Medical Entomology & Tropical Medicine (LEMMT), Colegio de Ciencias Biológicas y Ambientales (COCIBA), Universidad San Francisco de Quito (USFQ), Quito 170901, Ecuador; rleon@usfq.edu.ec; 3 Department of Microbiology, Faculty of Veterinary Medicine and Zootechnics, Universidad de Guayaquil, Guayaquil 090514, Ecuador; 4 Institute of Genetics and Microbiology, Miguel Lillo Foundation, San Miguel de Tucumán T4000JFE, Argentina

**Keywords:** *Ancylostoma* spp., canine hookworms, Ecuador, One Health, prevalence, systematic review

## Abstract

Hookworms are zoonotic helminths that cause intestinal diseases in pets and humans, as well as cutaneous larva migrans in the latter; therefore, they are of importance to animal and public health. Stray and domestic dogs play a significant role in the transmission of hookworm disease. This study examines the prevalence of *Ancylostoma* spp. infections in dogs in Ecuador through a systematic review and meta-analysis. Although based exclusively on canine data, the findings are discussed in the context of available evidence on zoonotic transmission and potential One Health implications. A total of 370 records were screened, of which 9 studies published between 2010 and 2025 met the PRISMA 2020 inclusion criteria. Using a random-effects model with Freeman–Tukey double arcsine transformation, the pooled prevalence was estimated at 32% (95% CI: 17–49%), with substantial heterogeneity across studies (I^2^ = 98.3%). Regional analysis revealed significantly higher prevalence in the Insular region (50%) and the Coastal region (43%), compared to the Sierra (Andean region) (12%). These findings indicate marked spatial variability in canine *Ancylostoma* infections across Ecuador, likely reflecting underlying environmental, epidemiological, and methodological differences. While this study does not directly assess human or environmental data, the observed patterns are consistent with epidemiological conditions that may favor zoonotic transmission. Overall, this study highlights the need for integrated approaches consistent with a One Health perspective, including improved surveillance, veterinary interventions, and further research incorporating human and environmental components to better understand the epidemiology and potential zoonotic implications of hookworm infections.

## Simple Summary

Hookworm infection, caused by several species of *Ancylostoma*, is prevalent worldwide, especially in developing tropical and subtropical countries. There is limited information on the prevalence of *Ancylostoma* spp. in dogs in Ecuador. This research aimed to determine the prevalence and potential zoonotic relevance of *Ancylostoma* spp. infections in dogs in Ecuador through a systematic review and meta-analysis using a One Health approach. Ten studies published between 2010 and 2025 (370 records) were analyzed. A prevalence of 50% was established in the Insular region (Galapagos), 43% on the coast, and 12% in the Andean region. Our findings provide new information on the prevalence of these nematodes in dogs living in various parts of Ecuador and suggest the need for integrated approaches consistent with a One Health perspective to improve the prevention of this parasitic disease.

## 1. Introduction

Soil-transmitted helminths (STH) remain among the most widespread neglected infections globally, affecting more than 1.5 billion people [1] and contributing substantially to morbidity in low- and middle-income countries [2]. Within this group, zoonotic hookworms of the genus *Ancylostoma* represent an underrecognized but critical public health concern due to their complex transmission dynamics at the human–animal–environment interface [3,4].

Dogs play a central role as reservoirs of zoonotic hookworms, particularly *Ancylostoma caninum*, *A. braziliense*, and *A. ceylanicum*, which are widely distributed across tropical and subtropical regions [4,5]. These parasites sustain transmission cycles through environmental contamination: eggs shed in canine feces develop into infective larvae capable of penetrating human skin or being ingested [2,5]. As a result, canine populations function not only as hosts but also as amplifiers of environmental exposure risk.

In humans, exposure to infective larvae leads primarily to cutaneous larva migrans (CLM), a pruritic dermatological condition strongly associated with contaminated soils in tropical and coastal environments [2,6]. Although often self-limiting, CLM constitutes a sensitive indicator of environmental contamination and zoonotic transmission, reflecting the convergence of ecological, behavioral, and socioeconomic determinants [2,7].

Despite the global burden of hookworm infections, the epidemiology of zoonotic *Ancylostoma* spp. remains poorly characterized in many regions, particularly in Latin America. Transmission patterns are shaped by climatic conditions, sanitary infrastructure, canine population dynamics, and human–animal interactions, which vary widely within and between countries [8,9].

Ecuador represents a highly heterogeneous epidemiological setting where ecological diversity from tropical coastal zones to Andean and insular ecosystems, such as the Galápagos Islands, creates diverse conditions for parasite transmission. Warm and humid climates, the presence of free-roaming dog populations, and limitations in sanitation infrastructure contribute to the persistence of *Ancylostoma* spp. [2,10].

Several local studies have documented the presence of canine hookworms across different regions of the country; however, these investigations are highly heterogeneous in terms of diagnostic methods, sample size, study design, and geographic coverage [2,11]. In parallel, evidence in humans is largely restricted to sporadic reports of CLM, with limited integration between animal and human epidemiological data [6,12].

This fragmentation of evidence limits the ability to estimate the true burden of infection and to understand spatial and epidemiological patterns of transmission. In particular, no study to date has systematically synthesized the available data on the prevalence of *Ancylostoma* spp. in dogs at a national scale in Ecuador. Although focused exclusively on canine infections, this synthesis provides valuable epidemiological information that may contribute to future One Health investigations integrating animal, human, and environmental components. The findings describe infection patterns in canine populations and may serve as a foundation for future studies exploring human and environmental dimensions of hookworm transmission in Ecuador [10,13].

Therefore, this study aims to estimate the pooled prevalence of *Ancylostoma* spp. in dogs in Ecuador through a systematic review and meta-analysis following PRISMA 2020 guidelines [14]. Additionally, it seeks to evaluate heterogeneity among studies, identify regional differences in prevalence, and discuss potential zoonotic implications in light of the available literature.

## 2. Materials and Methods

A systematic review and meta-analysis were conducted to estimate the prevalence of *Ancylostoma* spp. in dogs in Ecuador. The review was designed to identify eligible studies, estimate pooled prevalence, and compare prevalence across the Coastal, Andean, and Insular regions of Ecuador. The study design, execution, and reporting followed the PRISMA 2020 guidelines for systematic reviews of observational studies [14]. No a priori protocol was developed for this review, and it was neither registered in PROSPERO nor in any other prospective registry; accordingly, no protocol amendments are applicable.

### 2.1. Eligibility Criteria

Eligibility criteria were defined a priori. Peer-reviewed studies were considered eligible if they were conducted in any province of Ecuador and included domestic or free-roaming dogs as the target population. Eligible designs comprised observational studies, particularly cross-sectional surveys or epidemiological sampling, reporting prevalence estimates of *Ancylostoma* spp. Diagnosis had to be based on standardized coproparasitological techniques, such as flotation, sedimentation, Baermann, Ritchie, or zinc sulfate methods, with molecular confirmation where available. Only studies published between 2010 and 2025 that provided sufficient quantitative data to estimate prevalence (i.e., number of positive cases and sample size) were included.

The time restriction (2010–2025) was established to maximize comparability among studies by focusing on contemporary epidemiological conditions and diagnostic methodologies. Older studies frequently used diagnostic approaches that differ substantially from those currently employed and may not accurately reflect current canine population dynamics in Ecuador.

Studies were excluded if they corresponded to grey literature or academic theses beyond the initial identification stage, case reports or clinical series without epidemiological design, studies based exclusively on environmental fecal samples without identifiable canine populations, or studies lacking quantitative prevalence data. Additionally, studies using unclear or non-standardized diagnostic methods, or those with non-eligible designs (e.g., clinical trials or narrative reviews), were excluded from the analysis.

### 2.2. Search Strategy

A comprehensive literature search was conducted in PubMed, Scopus, SciELO, Embase, and Google Scholar on 25 March 2026. Grey literature was screened to minimize publication bias and ensure comprehensive identification of potentially relevant studies; however, only peer-reviewed articles were retained for quantitative synthesis.

No language restrictions were applied; only studies published between 2010 and 2025 were considered eligible. This temporal restriction was applied to ensure comparability of diagnostic approaches and to reflect contemporary epidemiological conditions. However, this decision may introduce temporal selection bias, which is acknowledged as a limitation.

Search strings combined subject-specific terms with Boolean operators. An illustrative example of the strategy used in PubMed was: (“*Ancylostoma*” [MeSH] OR “*Ancylostoma caninum*” OR “hookworm” OR “canine hookworm”) AND (“Ecuador” OR “Galápagos”) AND (“prevalence” OR “epidemiology” OR “infection”). Equivalent strategies were adapted for each database using their respective controlled vocabularies (e.g., Emtree in Embase) in Spanish and English, including: “*Ancylostoma caninum*”, “canine hookworms”, “Ecuador”, “prevalence”, “cutaneous larva migrans”, “geohelminths”, and “dog parasites Ecuador”. No study-type filter was applied given the limited number of available studies in the region. Complete search strings for each database are provided in Supplementary Table S1.

### 2.3. Study Selection (PRISMA Process)

**Figure 1:**
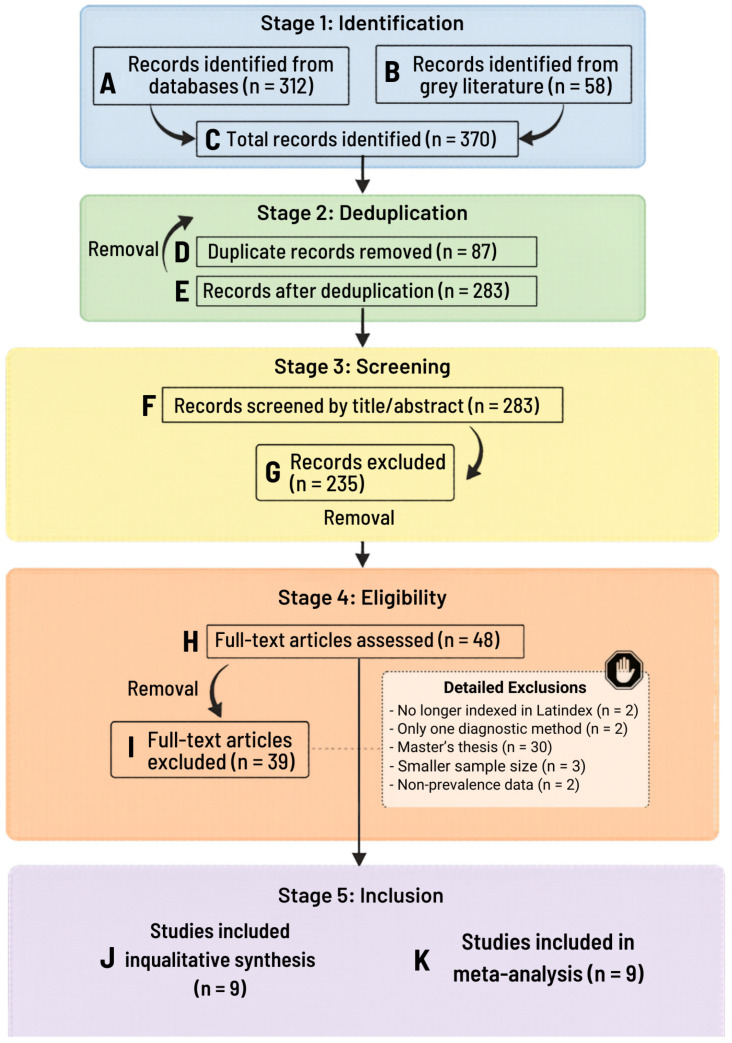
PRISMA 2020 flow diagram illustrating the study selection process.

The study selection process followed the PRISMA 2020 guidelines and is summarized in Figure 1. Two reviewers independently screened titles and abstracts, followed by independent full-text assessment of potentially eligible records. Discrepancies at any stage were resolved by discussion and consensus; when agreement was not reached, a third reviewer was consulted. Only studies meeting all inclusion criteria were retained for the final meta-analysis.

### 2.4. Data Extraction

Data were extracted using a standardized data collection framework specifically designed for this study. For each eligible article, information was systematically recorded, including the first author and year of publication, geographic location (province and region), characteristics of the canine population (owned or free-roaming), sample size, number of positive cases for *Ancylostoma* spp., diagnostic methods employed, and reported prevalence values.

Prevalence estimates were derived either directly from reported values or calculated as the proportion of positive cases relative to the total sample size.

Data extraction was performed independently by two reviewers. Discrepancies were resolved by consensus or adjudication by a third reviewer. When data were missing or ambiguous, the corresponding authors were contacted where feasible; otherwise, such studies were excluded from the affected analyses, and this was noted accordingly. All extracted data were compiled into a unified dataset and underwent a cross-verification process prior to statistical analysis.

For consistency, “owned dogs” were defined as dogs reported as having an identifiable owner regardless of their roaming behavior, whereas “free-roaming dogs” referred to dogs with unrestricted movement, irrespective of ownership status. When studies did not provide sufficient information to distinguish these categories, populations were classified as mixed.

Ownership status was extracted a priori because dog management practices are recognized determinants of helminth transmission and could potentially contribute to between-study heterogeneity. This variable was considered for subgroup analyses when sufficient data were available. However, statistically robust comparisons could not be performed due to the limited number of studies and the heterogeneity of reporting across publications.

### 2.5. Statistical Analysis

The meta-analysis was performed using a random-effects model based on the DerSimonian–Laird method [15], accounting for expected variability between studies.

The primary outcome was pooled prevalence. To stabilize variance and reduce bias associated with extreme proportions, prevalence estimates were transformed using the Freeman–Tukey double arcsine method and subsequently back-transformed for interpretation.

Due to the limited number of studies, meta-regression analyses were not performed. When studies reported only raw counts (positive cases and sample size) without a prevalence estimate, proportions were calculated directly. No additional data conversions were required.

### 2.6. Heterogeneity Assessment

Between-study heterogeneity was assessed using Cochran’s Q statistic and the I^2^ index, which quantifies the proportion of total variability attributable to true heterogeneity rather than sampling error [16,17]. In addition, between-study variance (τ^2^) and its standard deviation (τ) were calculated to provide absolute measures of dispersion among prevalence estimates.

### 2.7. Subgroup Analysis

A subgroup meta-analysis was conducted by geographic region (Coast, Andean, and Insular), applying the same statistical approach used in the global analysis.

For each subgroup, pooled prevalence and heterogeneity (I^2^) were estimated.

### 2.8. Prediction Intervals

Prediction intervals were calculated under the random-effects model to estimate the expected range of prevalence in future studies conducted under similar epidemiological conditions.

### 2.9. Graphical Analysis

Results were visualized using graphical approaches, including forest plots displaying individual and pooled prevalence estimates, density plots illustrating the distribution and dispersion of prevalence across studies, and a funnel plot to explore potential publication bias and error structure. The funnel plot provided a visual assessment of study effect sizes against their standard errors, complementing the heterogeneity metrics and ensuring that variability and bias were systematically evaluated.

### 2.10. Software

All statistical analyses were conducted in R version 4.4.2 (R Foundation for Statistical Computing, Vienna, Austria) using RStudio version 2024.12.1+563 (Posit Software, Boston, MA, USA). The meta package version 8.5-0 was used to estimate pooled prevalence, while the metafor package version 5.0-1 was applied for heterogeneity assessment, prediction intervals, publication bias analyses, and sensitivity analyses. Graphical outputs were generated using standard visualization functions within the R environment.

### 2.11. Risk of Bias Assessment

The risk of bias of the studies included was assessed using a qualitative approach based on the Newcastle–Ottawa Scale (NOS) domains for observational studies: selection of study groups, comparability of groups, and ascertainment of exposure or outcome. Two reviewers independently evaluated each study, and discrepancies were resolved by consensus. Studies were subsequently classified as low (≥7 points), moderate (5–6 points), or high risk of bias (≤4 points). Assessments are summarized in Supplementary Table S2.

### 2.12. Sensitivity Analysis

Sensitivity analyses were conducted using the metafor package (version 5.0-1) in R version 4.4.2 and RStudio version 2024.12.1+563, applying a leave-one-out approach in which the model was recalculated iteratively after excluding one study at a time.

### 2.13. Publication Bias Assessment

Publication bias was assessed by visual inspection of funnel plots combined with Egger’s regression test, implemented via the metafor package. A statistically significant intercept (*p* < 0.05) was interpreted as evidence of funnel plot asymmetry suggestive of publication bias.

### 2.14. Certainty of Evidence

The certainty of the evidence was assessed using the GRADE approach, considering risk of bias, inconsistency, imprecision, and potential publication bias. As all included studies were observational, the initial certainty was rated as low and adjusted based on these domains. The overall certainty was classified as high, moderate, low, or very low.

## 3. Results

The meta-analysis revealed a high and heterogeneous prevalence of *Ancylostoma* spp. in dogs in Ecuador, with marked variability across regions and study contexts. The main characteristics of the included studies are summarized in Table 1, including publication year, geographic location, sample size, number of positive cases, prevalence estimates with 95% confidence intervals, diagnostic methods, study design, dog population type, and sampling strategy. Most studies were cross-sectional and included mixed or free-roaming dog populations, with sampling strategies often not reported. Variability in diagnostic approaches was also observed across studies, and may partially explain the high heterogeneity detected in the meta-analysis. Studies contributing to each regional subgroup synthesis (Coastal, *n* = 4; Andean, *n* = 3; Insular, *n* = 2) were predominantly cross-sectional in design, with no subgroup dominated by a single diagnostic approach.

A total of 9 studies (Table 1) were included in the meta-analysis, comprising 2087 dogs, of which 762 tested positive for *Ancylostoma* spp., corresponding to a crude prevalence of 36.5%.

Using a random-effects model, the pooled prevalence of *Ancylostoma* spp. in dogs in Ecuador was estimated at 32% (95% CI: 17–49%). Substantial heterogeneity was observed among studies (I^2^ = 98.3%; τ^2^ = 0.0668; *p* < 0.0001), indicating considerable variability in prevalence estimates across study settings.

Substantial heterogeneity was observed among studies (I^2^ = 98.3%; τ^2^ = 0.0668; *p* < 0.0001), indicating pronounced variability in prevalence estimates across study settings.

**Figure 2:**
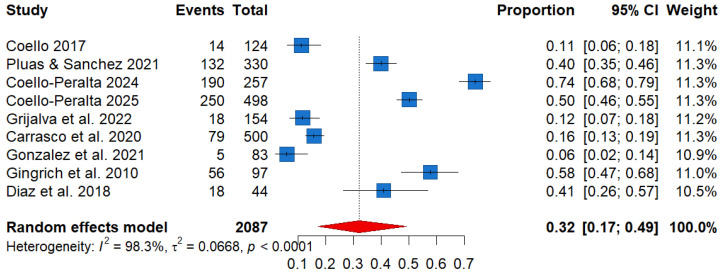
Forest plot of individual study prevalence estimates with 95% confidence intervals under a random-effects model. The diamond represents the pooled prevalence estimate, and the red line indicates the prediction interval. Heterogeneity statistics are shown at the bottom of the figure. Studies included in the analysis correspond to references [2,11,12,18,19,20,21,22,23].

The forest plot (Figure 2) illustrates the wide dispersion of prevalence estimates across studies, with multiple studies clustering at low and high values. Each study is represented by its point estimate and corresponding 95% confidence interval, while the dashed vertical line indicates the pooled prevalence under the random-effects model.

**Figure 3:**
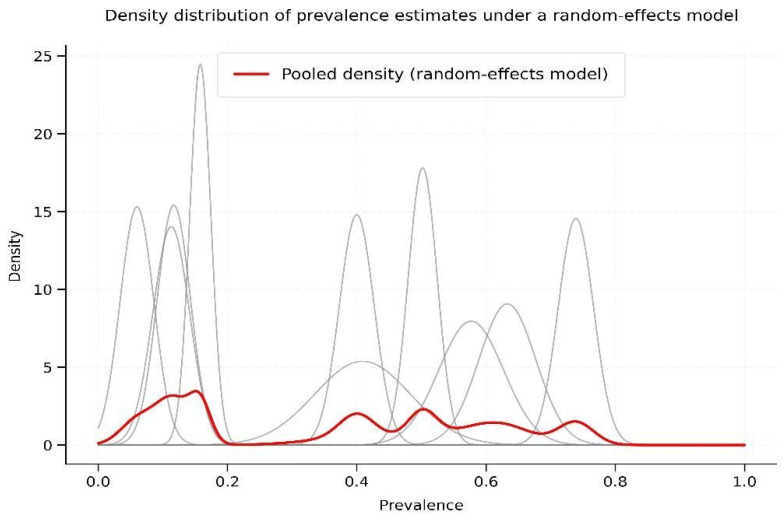
Density distribution of prevalence estimates under a random-effects model. Grey curves represent study-specific prevalence densities, while the red curve shows the pooled distribution under a random-effects model. The broad and multimodal pattern reflects substantial between-study heterogeneity.

The density plot (Figure 3) illustrates the distribution of prevalence estimates across studies, showing multiple peaks that indicate the presence of distinct clusters of values. The pooled distribution appears broader and flatter, reflecting the high level of heterogeneity captured by the random-effects model. This pattern is consistent with the high I^2^ value (>90%), which indicates that the observed variability is primarily driven by substantial epidemiological differences rather than random variation.

**Figure 4:**
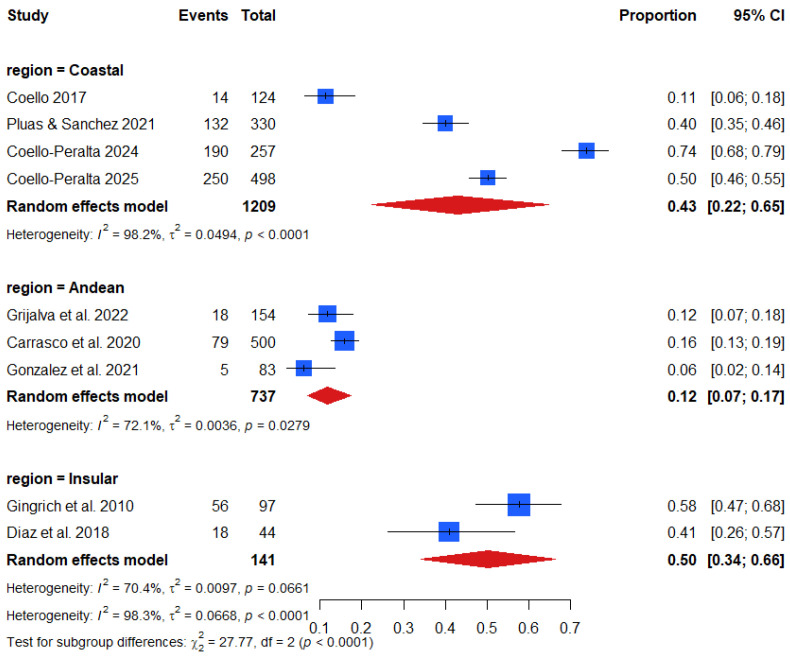
Forest plot of *Ancylostoma* spp. prevalence in dogs by geographic region in Ecuador under a random-effects model. Diamonds represent pooled prevalence estimates for each subgroup, and horizontal lines indicate 95% confidence intervals. Studies included in the subgroup analyses correspond to references [2,11,12,18,19,20,21,22,23].

Regional differences in prevalence were evident (Figure 4 and Table 2). The Coastal region showed a pooled prevalence of 43% (95% CI: 22–65%), with very high heterogeneity (I^2^ = 98.2%).

The Andean region presented a lower prevalence of 12% (95% CI: 7–17%; I^2^ = 72.1%), while the Insular region exhibited a prevalence of 50% (95% CI: 34–66%) with moderate-to-high heterogeneity (I^2^ = 70.4%).

Clear regional patterns emerged, with substantially higher prevalence in coastal and insular regions compared to Andean regions. Prediction intervals indicated a wide range of expected prevalence values for future studies (Table 3). At the national level, prevalence was estimated to range from approximately 2% to 83%, reflecting substantial variability between epidemiological contexts.

**Figure 5:**
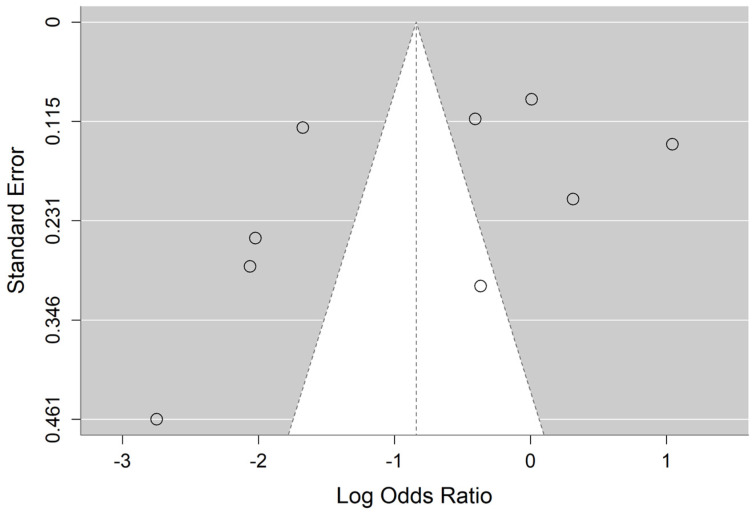
Funnel plot of the meta-analysis of *Ancylostoma* spp. prevalence studies in dogs in Ecuador. Each circle represents an individual study plotted according to its standard error and log odds ratio. The dashed vertical line represents the pooled effect estimate obtained under a random-effects model, and the diagonal lines indicate the expected 95% confidence limits in the absence of publication bias. The gray shaded area represents the region within which studies are expected to fall in the absence of publication bias.

Publication bias was evaluated using visual inspection of the funnel plot (Figure 5) combined with Egger’s regression test. Visual interpretation of funnel plot asymmetry should be made cautiously because only nine studies were included and substantial heterogeneity was observed across studies. Visual inspection revealed slight asymmetry, with fewer small studies reporting low prevalence values; however, the overall pattern remained broadly symmetrical around the pooled estimate. Egger’s test did not identify statistically significant asymmetry (*p* > 0.05), indicating no clear evidence of publication bias was detected; however, this assessment is limited by the small number of included studies. The mild dispersion at the lower precision end is consistent with the high heterogeneity observed (I^2^ = 98.3%), reflecting diverse epidemiological contexts and diagnostic methods across regions.

Studies excluded after full-text assessment, with the primary reason for exclusion (e.g., absence of quantitative prevalence data, non-eligible diagnostic methods, non-eligible study design), are listed in Supplementary Table S3.

Most included studies were cross-sectional and relied on conventional coproparasitological techniques. Studies incorporating multiple diagnostic approaches, particularly those including molecular confirmation (PCR), demonstrated greater methodological robustness and were classified as low risk of bias. Studies using single diagnostic methods or lacking detailed sampling frameworks were classified as moderate or high risk. Overall, the body of evidence was considered to present a moderate risk of bias, primarily related to limitations in sampling representativeness and control of confounding factors. Individual assessments are provided in Supplementary Table S2.

The leave-one-out sensitivity analysis confirmed that the pooled prevalence estimate was stable. Exclusion of any individual study did not substantially modify the overall findings, and no single study was identified as disproportionately influential. Results were consistent across fixed-effect and random-effects models, reinforcing the robustness of the reported epidemiological patterns.

The certainty of the evidence was rated as moderate according to the GRADE framework, based on variability in study designs, diagnostic methods, sample sizes, and geographic coverage observed across the included studies. Despite these limitations, the consistent detection of *Ancylostoma* spp. across regions supports the overall reliability of the findings. A structured Summary of Findings is provided in Table 4.

## 4. Discussion

The present meta-analysis provides the first integrated estimation of the prevalence of *Ancylostoma* spp. in dogs in Ecuador. Although the crude prevalence across all included studies was 36.5%, the random-effects meta-analysis yielded a pooled prevalence of 32% (95% CI: 17–49%), reflecting the substantial heterogeneity observed among studies [2,11,12,18,19,20,21,22,23]. This level of prevalence is consistent with findings from other tropical and subtropical regions, where environmental and socioecological conditions favour the persistence of soil-transmitted helminths. For instance, a large-scale meta-analysis in Asia reported a pooled prevalence of 41% (29–53%) in dogs [24], suggesting that the burden observed in Ecuador reflects broader global patterns associated with warm climates and environmental exposure.

At the regional level, marked differences in prevalence were observed, with higher values in the Coastal region (43%; 95% CI: 22–65%) and the Insular region (50%; 95% CI: 34–66%), compared to significantly lower values in the Andean region (12%; 95% CI: 7–17%). These findings are consistent with previous studies demonstrating that temperature, humidity, and soil characteristics are key determinants of hookworm transmission [8,9]. Tropical coastal environments and insular ecosystems provide optimal conditions for larval survival, while high-altitude regions typically limit parasite development due to lower temperatures and reduced environmental suitability [25,26].

The elevated prevalence observed in Insular region is particularly relevant from an epidemiological perspective. Unlike mainland regions, studies in the islands predominantly include free-roaming dog populations, which experience limited access to veterinary care and deworming interventions [21,27]. This context, combined with high environmental exposure and close interaction with humans and animals, may favor parasite transmission and warrants further investigation of its epidemiological significance.

The high level of heterogeneity observed across studies (I^2^ = 98.3%) suggests that the variability in prevalence estimates was driven primarily by true differences among study settings rather than by random error [28,29,30,31]. Several factors may have contributed to this heterogeneity, including geographic variation, differences in canine populations (owned, free-roaming, or mixed), and the use of different coproparasitological diagnostic techniques with varying sensitivities [32,33,34]. Although all included studies met the predefined eligibility criteria and provided extractable quantitative prevalence data, some were not originally designed as prevalence studies. Consequently, differences in study design, sampling frameworks, and data collection approaches may have further contributed to the observed variability and should be considered when interpreting the pooled estimates.

Diagnostic variability deserves particular attention because flotation, sedimentation, Baermann, and other coproparasitological methods differ in their sensitivity and specificity, potentially influencing prevalence estimates and contributing to between-study heterogeneity [29,30,31]. Consistent with this methodological variability, the risk-of-bias assessment (Supplementary Table S2) indicated that most studies presented a moderate risk of bias, mainly due to cross-sectional designs, limited representativeness, and the absence of standardized diagnostic protocols. Although differences among diagnostic approaches were evident, the limited number of studies and the unequal distribution of methods prevented robust comparisons of prevalence according to diagnostic technique.

Only one study incorporating PCR was classified as low risk, highlighting the importance of molecular confirmation for accurate prevalence estimation. The sensitivity analysis further confirmed the robustness of the pooled estimate: no single study disproportionately influenced the results, and findings were consistent across model specifications. Egger’s regression test did not identify statistically significant funnel plot asymmetry, and the GRADE assessment rated the overall certainty of evidence as moderate, reflecting methodological variability but consistent findings across studies.

From a One Health perspective, the findings of this meta-analysis gain additional relevance when considered alongside evidence of human infection. Reports of cutaneous larva migrans (CLM) have been documented in several Ecuadorian regions where canine *Ancylostoma* infections have also been reported, particularly in coastal and tropical environments. Although these observations suggest potential geographic overlap, no direct epidemiological association was assessed in the present study [2,6]. This spatial overlap is consistent with potential zoonotic interactions, although direct transmission was not assessed in this study, where environmental contamination acts as the interface between animal reservoirs and human exposure.

Evidence from molecular studies further reinforces this connection. High genetic similarity between *Ancylostoma* spp. detected in dogs and humans in Ecuador may suggest potential cross-transmission and shared epidemiological cycles [10]. Although most studies included in the meta-analysis reported findings at the genus level (*Ancylostoma* spp.), available molecular evidence from Ecuador suggests that *A. caninum* is the predominant species infecting dogs, while *A. ceylanicum* has also been detected in humans and represents an important zoonotic concern. The presence of these species supports the epidemiological relevance of canine populations as potential reservoirs of zoonotic hookworms and highlights the need for future studies incorporating molecular characterization to better define species distribution and transmission dynamics in Ecuador [3,10,12].

Similar patterns have been reported globally, including the detection of zoonotic hookworms such as *A. ceylanicum* in both canine and human populations [32,33], emphasizing the need for integrated surveillance systems that address both animal and human health.

Previous studies have suggested that socioeconomic and management factors may influence infection patterns; however, these variables were not evaluated in the present meta-analysis. Studies have consistently shown higher prevalence in stray or free-roaming dogs compared to owned animals, primarily due to reduced access to veterinary care and deworming programs [34]. In Latin America and similar settings, disparities in sanitation and access to veterinary services contribute to sustained transmission cycles, reinforcing the importance of addressing structural determinants of disease [35,36,37].

The absence of eligible studies from the Amazon region represents an important gap in the available evidence. Therefore, no conclusions can be drawn regarding the epidemiology of canine *Ancylostoma* spp. in this region.

Consequently, the lack of data should not be interpreted as absence of infection, but rather as a limitation in surveillance and research coverage.

The wide prediction intervals observed in this study further emphasize the variability in prevalence across epidemiological contexts. While the pooled estimate provides a useful summary measure, prediction intervals highlight that prevalence in future studies may vary substantially depending on local conditions. Therefore, the pooled prevalence should be interpreted cautiously, as it represents an average across highly heterogeneous epidemiological contexts and diagnostic approaches.

This reinforces the interpretation of the pooled estimate as an average of heterogeneous scenarios rather than a universal value.

Several limitations should be considered when interpreting these findings. The relatively small number of eligible studies, the heterogeneity in diagnostic methods may affect the precision and comparability of prevalence estimates. Finally, this review was not prospectively registered in PROSPERO or any other public registry prior to data collection, which may limit the verifiability of protocol decisions. Nevertheless, all methodological decisions were defined a priori and reported in full accordance with PRISMA 2020 guidelines. These findings should be interpreted within the limitations of the available data and highlight the need for more standardized and integrative epidemiological studies.

Despite these limitations, this study provides a robust synthesis of available evidence and highlights key epidemiological patterns of *Ancylostoma* spp. in Ecuador. The findings underscore the need for integrated control strategies under a One Health approach, including regular deworming of dog populations, responsible dog ownership programs, and the development of coordinated animal–human surveillance systems.

## 5. Conclusions

This meta-analysis demonstrates that *Ancylostoma* spp. infection is common in dogs in Ecuador, with substantial regional variability across the included studies. The high prevalence observed, particularly in coastal and insular regions, highlights the importance of canine populations as reservoirs of infection.

Although this study is based exclusively on canine data, the observed patterns are consistent with conditions that may be relevant from a zoonotic perspective and warrant further investigation through studies integrating animal, human, and environmental data. However, direct evidence of zoonotic transmission was not assessed and should be interpreted with caution.

These findings underscore the need for strengthened surveillance and control strategies focused on canine populations, as well as further studies integrating human and environmental data to better understand the broader epidemiological context. Future research should prioritize underrepresented regions, such as the Amazon, and incorporate standardized methodologies and molecular tools to refine prevalence estimates and improve comparability across studies.

## Figures and Tables

**Table 1:** Characteristics of included studies and prevalence estimates of *Ancylostoma* spp. in dogs in Ecuador.

Author (Year)	Region	Study Design	Dog Population	Sampling Strategy	*n*	Positive Cases	Prevalence (95% CI)	Diagnostic Method	Notes
Coello et al. 2017 (*ESPAMCIENCIA*) [18].	Coast	Cross-sectional	Owned	Not reported	124	14	11.3% (6.8–18.1)	F + B (coproparasitological)	Only canine data extracted
Pluas & Sánchez 2021 (*Bol. Malariol. Salud Ambient*) [19].			Mixed		330	132	40.0% (34.9–45.4)	F + S (coproparasitological)	
Coello et al. 2024 (*Med. Sci. Monit)* [2].			Mixed		257	190	73.9% (68.2–78.9)	D + F + S + B	
Coello et al. 2025 (*Sci. Rep)* [12].			Mixed		498	250	50.2% (45.8–54.6)	F + S + B + PCR	
Grijalva et al. 2022 (*JSMCAH*) [11].	Andean		Owned		154	18	11.7% (7.5–17.7)	D + F + S	
Carrasco et al. 2020 (*PJAEE)* [20].			Owned		500	79	15.8% (12.9–19.3)	D + ZnF	
González et al. 2021 (*Vet. Parasitol. Reg. Stud. Reports*) [21].			Mixed		83	5	6.0% (2.6–13.3)	R	
Gingrich et al. 2010 (*Vet. Parasitol)* [22].	Insular		Mixed		97	56	57.7% (47.8–67.1)	F	
Díaz et al. 2018 (*J. Prev. Vet. Med*) [23].			Free-roaming		44	18	40.9% (27.7–55.6)	D + F + S	

Abbreviations: Authors and Journal; F = flotation; S = sedimentation; R = Ritchie; B = Baermann; D = direct smear; ZnF = zinc sulfate flotation; PCR = polymerase chain reaction. Note: Only studies reporting extractable prevalence data (number of positive cases and sample size) in canine populations were included. Studies with mixed objectives were included only when canine-specific prevalence estimates could be clearly derived.

**Table 2:** Pooled prevalence and heterogeneity estimates of *Ancylostoma* spp. in dogs by geographic region in Ecuador.

Region	Studies (*n*)	Pooled Prevalence	I^2^ (%)	Interpretation of Heterogeneity
Coastal	4	43% (22–65%)	98.2%	Very high
Andean	3	12% (7–17%)	72.1%	High
Insular	2	50% (34–66%)	70.4%	Moderate–high

**Table 3:** Prediction intervals and pooled prevalence of *Ancylostoma* spp. in dogs by geographic region in Ecuador.

Region	Studies (*n*)	Pooled Prevalence	Prediction Interval
Coastal	4	~43%	≈4–87%
Andean	3	~12%	≈2–36%
Insular	2	~50%	≈18–81%
Overall	9	~32%	≈2–83%

Note: Pooled prevalence values correspond to random-effects meta-analysis estimates and differ from the crude proportion of positive animals across all included studies.

**Table 4:** Summary of Findings (GRADE assessment of evidence certainty).

Outcome	No. of Studies	Study Design	Risk of Bias	Inconsistency	Imprecision	Publication Bias	Overall Certainty
*Ancylostoma* spp. prevalence in dogs	9	Observational	Moderate	Moderate	Low	Low	Moderate

## Data Availability

The datasets used and analysed during the current study are available from the corresponding author upon reasonable request.

## Supplementary Materials

The following supporting information can be downloaded at: https://www.mdpi.com/article/10.3390/ani16142230/s1. File S1. PRISMA_2020_checklist. Table S1. Search strings used in each database. Table S2. Qualitative risk of bias assessment of included cross-sectional studies based on methodological characteristics reported in the main manuscript. Table S3. Studies excluded after full-text assessment with reasons for exclusion.
