# Supplementary material for: High Prevalence and Regional Heterogeneity of Canine Ancylostoma spp. in Ecuador: A Systematic Review and Meta-Analysis and Its Potential One Health Implications
Source: Animals (Basel). 2026 Jul 18;16(14):2230. doi: 10.3390/ani16142230 (PMC13404796; doi:10.3390/ani16142230)
Supplement: Supplementary file 1 [file animals-16-02230-s001.zip › Supplementary_Table_S1.pdf]

Supplementary Materials

High prevalence and regional heterogeneity of canine Ancylostoma spp. in Ecuador: a systematic review and meta-analysis from a One Health perspective

Vinueza et al.

Table S1. Search strings used in each database.

Database	Search string	Filters applied	Notes
PubMed	("Ancylostoma"[MeSH Terms] OR "Ancylostoma caninum"[tiab] OR "hookworm"[tiab] OR "canine hookworm"[tiab] OR "Uncinaria stenocephala"[tiab] OR "geohelminths"[tiab]) AND ("Ecuador"[tiab] OR "Galápagos"[tiab] OR "Galapagos"[tiab]) AND ("prevalence"[tiab] OR "epidemiology"[tiab] OR "infection"[tiab] OR "parasitosis"[tiab] OR "cutaneous larva migrans"[tiab])	Years: 2010–2025; Language: none; Study type: none	Used MeSH terms + free-text [tiab]
Scopus	(TITLE-ABS-KEY("Ancylostoma" OR "Ancylostoma caninum" OR "hookworm" OR "canine hookworm" OR "Uncinaria stenocephala" OR "geohelminths") AND TITLE-ABS-KEY("Ecuador" OR "Galapagos" OR "Galápagos") AND TITLE-ABS-KEY("prevalence" OR "epidemiology" OR "infection" OR "parasitosis" OR "cutaneous larva migrans") AND PUBYEAR > 2009 AND PUBYEAR < 2026)	Years: 2010–2025; Language: none; Study type: none	TITLE-ABS-KEY field tag; year filter via PUBYEAR
Embase	('Ancylostoma'/exp OR 'Ancylostoma caninum' OR 'hookworm' OR 'canine hookworm' OR 'Uncinaria stenocephala' OR 'geohelminths') AND ('Ecuador' OR 'Galapagos') AND ('prevalence' OR 'epidemiology' OR 'infection' OR 'parasitosis' OR 'cutaneous larva migrans') AND [2010-2025]/py	Years: 2010–2025; Language: none; Study type: none	Emtree term (/exp) + free text; [py] year filter
SciELO	(ti:(Ancylostoma OR "hookworm" OR "helmintos" OR "parásitos caninos" OR "geohelmintos") AND ti:(Ecuador OR Galápagos) OR ab:(Ancylostoma OR hookworm OR helmintos OR "parásitos intestinales") AND ab:(Ecuador OR Galápagos) AND ab:(prevalencia OR epidemiología OR prevalence OR epidemiology))	Years: 2010–2025; Language: none; Study type: none	ti: (title) and ab: (abstract) field tags
Google Scholar	"Ancylostoma" OR "Ancylostoma caninum" OR "hookworm" OR "geohelminths" AND "Ecuador" OR "Galápagos" AND "prevalence" OR "prevalencia" OR "epidemiology" OR "epidemiología"	Years: 2010–2025 (custom range); Language: none; Study type: none	Free-text only; no controlled vocabulary; first 200 results screened
Institutional repositories (grey literature)	"Ancylostoma" OR "Ancylostoma caninum" OR "uncinariasis" AND "Ecuador" OR "perros" OR "caninos". Searched in: USFQ repository, Universidad Central del Ecuador, and other Ecuadorian university repositories.	No date filter; Spanish only; Theses & reports	Grey literature; theses excluded from final analysis per eligibility criteria

Abbreviations: MeSH = Medical Subject Headings (PubMed); Emtree = Embase controlled vocabulary (/exp = exploded term); tiab = title/abstract field tag (PubMed); TITLE-ABS-KEY = title, abstract, and keyword field tag (Scopus); ti = title field (SciELO); ab = abstract field (SciELO); PUBYEAR = publication year filter (Scopus); [py] = publication year filter (Embase). Equivalent free-text terms in Spanish were applied in SciELO and grey literature searches.
