# Supplementary material for: High Prevalence and Regional Heterogeneity of Canine Ancylostoma spp. in Ecuador: A Systematic Review and Meta-Analysis and Its Potential One Health Implications
Source: Animals (Basel). 2026 Jul 18;16(14):2230. doi: 10.3390/ani16142230 (PMC13404796; doi:10.3390/ani16142230)
Supplement: Supplementary file 1 [file animals-16-02230-s001.zip › Supplementary_Table_S2.pdf]

Supplementary Materials

High prevalence and regional heterogeneity of canine Ancylostoma spp. in Ecuador: a systematic review and meta-analysis from a One Health perspective

Vinueza et al.

Table S2. Qualitative risk of bias assessment of included cross-sectional studies based on methodological characteristics reported in the main manuscript.

Study (Author, Year)	Diagnostic approach	Methodological limitations	Overall risk of bias
Coello 2019	F + B	Limited representativeness, single location	Moderate
Coello 2017	F + B	Small number of positives, limited comparability	Moderate
Pluas & Sánchez 2021	F + S	No molecular confirmation	Moderate
Coello-Peralta 2024	D + F + S + B	No explicit confounder control	Moderate
Coello-Peralta 2025	F + S + B + PCR	Strong methodology, multiple diagnostics	Low
Grijalva et al. 2022	D + F + S	Limited representativeness	Moderate
Carrasco et al. 2020	D + ZnF	Lack of multiple diagnostic methods	Moderate
González et al. 2021	R	Single diagnostic approach, low sensitivity	High
Gingrich et al. 2010	F	No multiple methods, older study design	Moderate
Díaz et al. 2018	D + F + S	Small sample size	Moderate

Qualitative assessment of risk of bias in the included studies based on study design, diagnostic approach, and methodological characteristics reported in Table 1 of the main manuscript. All studies were observational and primarily cross-sectional. Risk of bias was categorized as low, moderate, or high according to the likelihood of selection bias, adequacy of diagnostic methods, and control of confounding factors. Studies incorporating multiple diagnostic techniques, including molecular methods (e.g., PCR), were considered to have lower risk of bias. Abbreviations: F = flotation; S = sedimentation; B = Baermann; D = direct smear; ZnF = zinc sulfate flotation; PCR = polymerase chain reaction; R = Ritchie.
