# Supplementary material for: High Prevalence and Regional Heterogeneity of Canine Ancylostoma spp. in Ecuador: A Systematic Review and Meta-Analysis and Its Potential One Health Implications
Source: Animals (Basel). 2026 Jul 18;16(14):2230. doi: 10.3390/ani16142230 (PMC13404796; doi:10.3390/ani16142230)
Supplement: Supplementary file 1 [file animals-16-02230-s001.zip › Supplementary_Table_S3.pdf]

Supplementary Materials

High prevalence and regional heterogeneity of canine Ancylostoma spp. in Ecuador: a systematic review and meta-analysis from a One Health perspective

Vinueza et al.

Table S3. Studies excluded after full-text assessment with reasons for exclusion.

Author (Year)	Title (abbreviated)	Stage excluded	Reason for exclusion
Paredes et al., 2021	Prevalencia de Ancylostoma sp. en perros domésticos de Babahoyo. Los Ríos, Ecuador.	Full-text	Article no longer indexed in Latindex
Calvopiña et al., 2023	Diversity and prevalence of gastrointestinal helminths of free-roaming dogs on coastal beaches in Ecuador	Full-text	Sample size exceeds specified limit
Moreno, D. & Mena, R., 2017	Endoparasitosis en caninos de dos localidades de la costa ecuatoriana.	Full-text	Bachelor's thesis
Sánchez-Palomino et al., 2021	Prevalencia de parásitos intestinales en perros domésticos de Babahoyo.	Full-text	Article no longer indexed in Latindex
Granda, D. & Bueno, M., 2018	Zoonosis parasitaria intestinal en el sector de las Piñas. Milagro, Ecuador.	Full-text	Bachelor's thesis
Catagña, R. & Toro, B., 2020	Prevalencia de parásitos intestinales en perros domésticos de Quito, Ecuador.	Full-text	Bachelor's thesis
Falcón, M. & Salas, M., 2019	Prevalencia de parásitos zoonóticos en perros de Cuenca.	Full-text	Bachelor's thesis
Bonilla, C., 2015	Prevalencia de Ancylostoma caninum en perros de Riobamba.	Full-text	Bachelor's thesis
Corte, V., 2018	Prevalencia de parásitos intestinales en perros de Riobamba.	Full-text	Bachelor's thesis
Ramón, G.., 2012	Prevalencia de Ancylostoma caninum en perros de Cuenca.	Full-text	Bachelor's thesis
Sarabia, A. & Armas, J., 2019	Prevalencia de Ancylostoma caninum en perros de Latacunga, Ecuador.	Full-text	Bachelor's thesis
Tuasa, C., 2015	Prevalencia de parásitos intestinales en perros de Ambato.	Full-text	Bachelor's thesis
Tobar K, 2023	Prevalencia de endoparásitos en caninos de Azogues.	Full-text	Bachelor's thesis
Basantes, J. & Salas, M., 2021	Prevalencia de Ancylostoma caninum en perros de Francisco de Orellana.	Full-text	Bachelor's thesis
Zhunio, M. & Salas, M., 2022	Prevalencia de parásitos intestinales en perros de Morona Santiago.	Full-text	Bachelor's thesis
Jaramillo, A., 2022	Prevalencia de Ancylostoma caninum en perros de Pastaza.	Full-text	Bachelor's thesis
Ube et al., 2023	Actualización de parasitosis en caninos, zona urbana, ciudad de Guayaquil.	Full-text	Single diagnostic method
Pérez, J. & Vera, F., 2025	Prevalencia de Ancylostoma caninum en perros domésticos de Calceta.	Full-text	Bachelor's thesis
Tinoco, G. & Salas, M., 2022	Prevalencia de parásitos zoonóticos en caninos de Cuenca.	Full-text	Master's thesis

Author (Year)	Title (abbreviated)	Stage excluded	Reason for exclusion
Calvopiña et al., 2024	Anthroponotic and Zoonotic Hookworm in dogs and humans from Ecuador.	Full-text	Smaller sample size
Neira, D., 2024	Prevalencia de parásitos gastrointestinales en perros de Guayaquil.	Full-text	Bachelor's thesis
Tierra-Carrasco et al., 2024	Epidemiología de Parásitos Gastrointestinales en Caninos de Riobamba.	Full-text	Smaller sample size
Aspiazu et al., 2022	Prevalencia de Ancylostoma caninum en perros de Vinces.	Full-text	Bachelor's thesis
Sacoto, M. & Salas, M., 2023	Prevalencia de parásitos intestinales en perros de Azogues.	Full-text	Bachelor's thesis
López, W., 2018	Prevalencia de Ancylostoma spp. y Toxocara spp. en caninos del recinto Puente Lucía, provincia del Guayas.	Full-text	Bachelor's thesis
Gómez, B., 2023	Prevalencia de Helmintos intestinales en perros de Lomas de Sargentillo.	Full-text	Bachelor's thesis
Lozano, S., 2015	Parásitos gastrointestinales en perros atendidos en un consultorio veterinario de Guayaquil.	Full-text	Bachelor's thesis
Cuesaca, A., 2025	Prevalencia de parásitos gastrointestinales en caninos del cantón Saquisilí.	Full-text	Bachelor's thesis
Meza, J. & Yagual, K., 2023	Prevalencia de parásitos intestinales zoonóticos en el parque Samanes de Guayaquil, Ecuador.	Full-text	Bachelor's thesis
Fogacho, N. & Pesántez, B., 2025	Presencia de parásitos gastrointestinales en caninos afectados con parvovirosis.	Full-text	Bachelor's thesis
Márquez, N., 2014	Prevalencia de parásitos gastrointestinales en caninos de la ciudad de Pasaje.	Full-text	Bachelor's thesis
Pombar, A., 2017	Prevalencia de protozoarios gastrointestinales en perros y gatos de dos refugios ubicados en la ciudad de Guayaquil.	Full-text	Bachelor's thesis
Coello et al., 2024	Impacto de los parásitos gastrointestinales en la salud animal y pública.	Full-text	Article on impact of gastrointestinal parasites on animal and public health; not a prevalence study
Velásquez et al., 2025	Prevalencia de parásitos intestinales en un refugio canino de Riobamba.	Full-text	Smaller sample size
Mejía, D., 2019	Parásitos gastrointestinales en perros atendidos en una clínica veterinaria de Guayaquil.	Full-text	Bachelor's thesis
Calle et al., 2023	Presencia de nematodos gastrointestinales zoonóticos en perros del Triunfo.	Full-text	Single diagnostic method
Segovia, I., 2020	Prevalencia de Parásitos intestinales en caninos de Quito.	Full-text	Bachelor's thesis
Chango, M., 2022	Presencia de parásitos gastrointestinales en caninos de la ciudad de Milagro.	Full-text	Bachelor's thesis
Coello et al., 2019	A Case of Cutaneous Larva Migrans in a Child from Vinces, Ecuador	Full-text	Non-prevalence data

Studies excluded after full-text assessment, with the primary reason for exclusion. All exclusions were based on pre-defined eligibility criteria: absence of quantitative prevalence data, non-eligible diagnostic methods (single method only), non-eligible study design (Master's theses, narrative reviews), or indexing issues.
